# Anti-neutrophil cytoplasmic antibody-associated glomerulonephritis with detection of myeloperoxidase and phospholipase A_2_ receptor in membranous nephropathy-lesions: report of two patients with microscopic polyangiitis

**DOI:** 10.1186/s12882-018-0922-5

**Published:** 2018-05-23

**Authors:** Kenta Tominaga, Takahiro Uchida, Toshihiko Imakiire, Kenji Itoh, Hideyuki Shimazaki, Kuniaki Nakanishi, Hiroo Kumagai, Naoki Oshima

**Affiliations:** 1 Department of Nephrology and Endocrinology, National Defense Medical College, 3-2 Namiki, Tokorozawa, Saitama 359-8513 Japan; 2 Division of Hematology and Rheumatology, Department of Internal Medicine, National Defense Medical College, Tokorozawa, Saitama Japan; 3 Department of Laboratory Medicine, National Defense Medical College, Tokorozawa, Saitama Japan

**Keywords:** Anti-neutrophil cytoplasmic antibody, Membranous nephropathy, Myeloperoxidase, Phospholipase A_2_ receptor

## Abstract

**Background:**

Podocyte phospholipase A_2_ receptor (PLA_2_R) is a major target antigen in idiopathic adult membranous nephropathy (MN). Histological PLA_2_R staining in the renal tissue has proven to be useful for the detection of idiopathic MN. However, glomerular PLA_2_R deposits have also been recently observed in several patients with secondary MN, such as hepatitis B virus-associated, hepatitis C virus-associated, and neoplasm-associated MN. Certain inflammatory environments have been suggested to lead to abnormal expression of PLA_2_R epitopes, with the resulting production of PLA_2_R autoantibodies.

**Case presentation:**

We report two patients diagnosed with anti-neutrophil cytoplasmic antibody (ANCA)-associated glomerulonephritis with MN-lesions, in whom ANCA titers for myeloperoxidase (MPO) were persistently positive. The first patient was a 52-years-old man who presented with interstitial pneumonitis. Microscopic hematuria and proteinuria were found when the interstitial pneumonitis became more severe. Renal biopsy findings yielded a diagnosis of ANCA-associated glomerulonephritis (mixed class) with MN-lesions. The second patient was a 63-years-old woman who had been treated for relapsing polychondritis. Her renal tissue showed evidence of focal ANCA-associated glomerulonephritis with MN-lesions. Interestingly, both MPO and PLA_2_R were detected in the glomerular subepithelial deposits of both patients. Immunoglobulin G (IgG) 1 and IgG2 were positive in the glomeruli of patient 2, and all subclasses of IgGs were positive in patient 1.

**Conclusion:**

The present cases suggest that ANCA-associated glomerulonephritis could expose PLA_2_R, leading to the development of MN-lesions.

## Background

In 2009, podocyte phospholipase A_2_ receptor (PLA_2_R) was reported as a major target antigen in idiopathic adult membranous nephropathy (MN) [1]. Subsequently, the presence of PLA_2_R antibodies in the serum has been shown to have high sensitivity and specificity for differentiating idiopathic MN from secondary MN [2]. In addition, histological PLA_2_R staining in renal tissue has been shown to be equally useful for the detection of idiopathic MN [3]. However, glomerular PLA_2_R deposits have also been observed in several patients with secondary MN [4]. For example, 64% of patients with hepatitis B virus (HBV)-associated MN were positive for renal PLA_2_R, overlapping with hepatitis B surface (HBs) antigen [5].

MN rarely occurs as a complication of anti-neutrophil cytoplasmic antibody (ANCA)-associated glomerulonephritis, and the pathological processes of the two diseases are generally thought to occur concurrently [6]. However, the pathogenesis of such disease and the involvement of PLA_2_R remain unclear.

We herein report two patients with microscopic polyangiitis (MPA) in whom ANCA-associated glomerulonephritis with MN-lesions developed. Although the levels were low, the ANCA titers for MPO were persistently positive in both patients. Interestingly, MPO and PLA_2_R were both detected in the glomerular subepithelial deposits of the two patients.

## Case presentation

### Patient 1

A 52-years-old man showing worsening of interstitial pneumonitis and presenting with microscopic hematuria and proteinuria was referred to our department. His interstitial pneumonitis was diagnosed 11 years ago, and since then, he had shown persistent serological positivity for MPO-ANCA. MPA was therefore suspected, and he was carefully followed-up without any medications.

After the referral, his proteinuria progressed to nephrotic syndrome. Physical examination showed bilateral fine crackles and pitting edema in the feet. His urinary protein excretion was 15.9 g/g urinary creatinine. Urinary microscopic examination showed massive erythrocytes. The results of blood examination were as follows: white blood cell count, 12.4 × 10^3^/μL; hemoglobin, 13.5 g/dL; platelet count, 529 × 10^3^/μL; serum creatinine, 1.99 mg/dL; urea nitrogen, 17 mg/dL; total protein/albumin (TP/Alb), 6.4/2.5 g/dL; total cholesterol, 245 mg/dL; immunoglobulin G (IgG), 1103 mg/dL; and IgA/M, 416/89 mg/dL. The C-reactive protein level was 1.2 mg/dL, and hypocomplementemia was absent. The ANCA titer for MPO was 19.4 U/mL, and the proteinase 3 (PR3) titer was within the normal range. Viral antibodies for HBV, HCV, and human immunodeficiency virus (HIV) were negative. His chest X-ray suggested exacerbation of interstitial pneumonitis. Computed tomography scans did not show any evidence of malignant tumors.

**Fig. 1:**
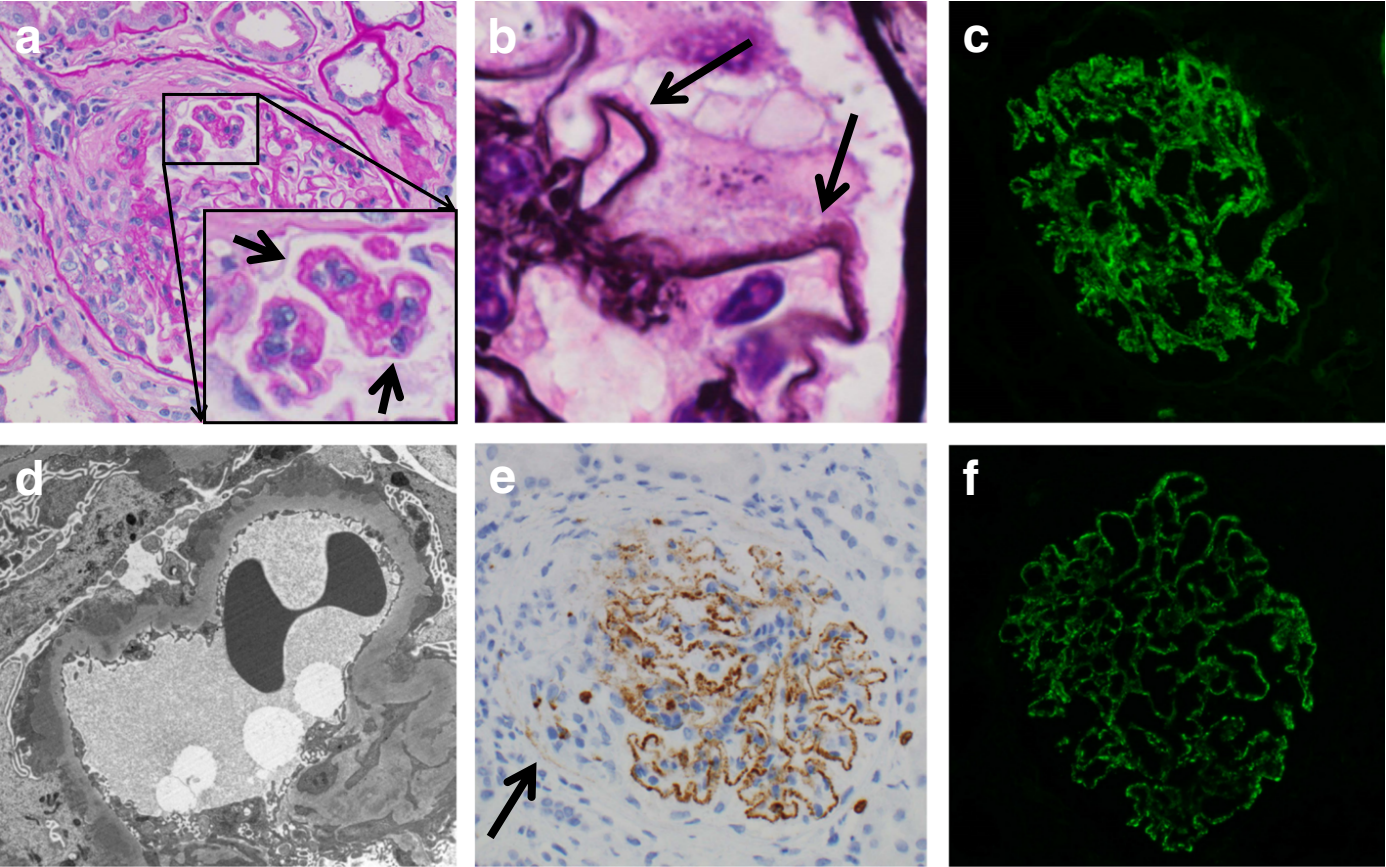
Histological features of the renal biopsy in patient 1. **a** Cellular crescent and endocapillary proliferation in the glomerulus by light microscopy (Periodic acid-Schiff staining). Duplication of the glomerular capillary walls was also suggested where prominent endocapillary proliferation was detected (arrows in inset). **b** Spike formation of the glomerular basement membrane (arrows) (Periodic acid silver-methenamine stain). **c** Positive immunofluorescence staining for immunoglobulin G on glomerular capillary walls. **d** Subepithelial electron-dense deposits, spike formation of the glomerular basement membrane, and effacement of the podocyte foot processes were shown on electron microscopy. **e** Immunoperoxidase staining for myeloperoxidase (MPO; Nichirei Biosciences, Tokyo, Japan) on paraffin-embedded tissue showed that the glomerular capillary walls and infiltrating neutrophils were positive for MPO. MPO staining was negative where the crescent was formed (arrow). **f** Indirect immunofluorescence staining for phospholipase A_2_ receptor (PLA_2_R; Sigma-Aldrich, St. Louis, MO) labeled with Alexa Fluor 488 (Thermo Fisher Scientific, Waltham, MA) on frozen tissue. The glomerular capillary walls were positive for PLA_2_R. Original magnification: **a**, **c**, **e**, **f**: 200×; **b**: 1000 ×

We diagnosed the patient with MPA clinically, and renal biopsy was performed. Light microscopy observations showed crescents in 13 of 28 glomeruli (Fig. 1a) as well as global glomerulosclerosis in 4 of 28 glomeruli. The biopsy also showed diffuse and global spike formation of the glomerular capillary walls (Fig. 1b). Immunofluorescence staining showed granular 2+ deposition of IgG (Fig. 1c) and complement C3 and ± deposition of IgM and complement C1q on the glomerular capillary walls. Electron microscopy showed subepithelial electron-dense deposits, spike formation of the glomerular basement membrane throughout the deposits, and effacement of the podocyte foot processes (Fig. 1d).

All of the IgG subclasses were positive on the glomerular capillary walls. Immunoperoxidase staining showed that the glomerular capillary walls were positive for MPO (Fig. 1e). In addition, although serum anti-PLA_2_R antibody level without any medications, measured by enzyme-linked immunosorbant assay at the day of renal biopsy, was negative, glomerular PLA_2_R was clearly detected (Fig. 1f). PLA_2_R staining was completely negative in the negative control (without the primary antibody, data not shown). According to these findings, the patient was diagnosed with ANCA-associated glomerulonephritis (mixed class) [7] with MN-lesions.

### Patient 2

A 63-years-old woman who had been treated with steroid therapy for relapsing polychondritis for 11 years was referred to our department because of continuous hematuria and proteinuria. The hematuria appeared 5 years ago, and the proteinuria appeared 1 year ago. Persistently positive ANCA titers for MPO were detected in her serum for 10 years.

Laboratory test findings were as follows: serum creatinine, 0.73 mg/dL; urea nitrogen, 8 mg/dL; TP/Alb, 6.6/4.4 g/dL; IgG, 793 mg/dL; and IgA/M, 208/115 mg/dL. Levels of complements and C-reactive protein were within the normal ranges. The ANCA titer for MPO was 25.6 U/mL, and the PR3 titer was negative. Viral antibodies for HBV, HCV, and HIV were negative. Urinalysis showed urinary protein excretion of 1.6 g/g urinary creatinine, and 10–19 erythrocytes were detected per high-power field.

**Fig. 2:**
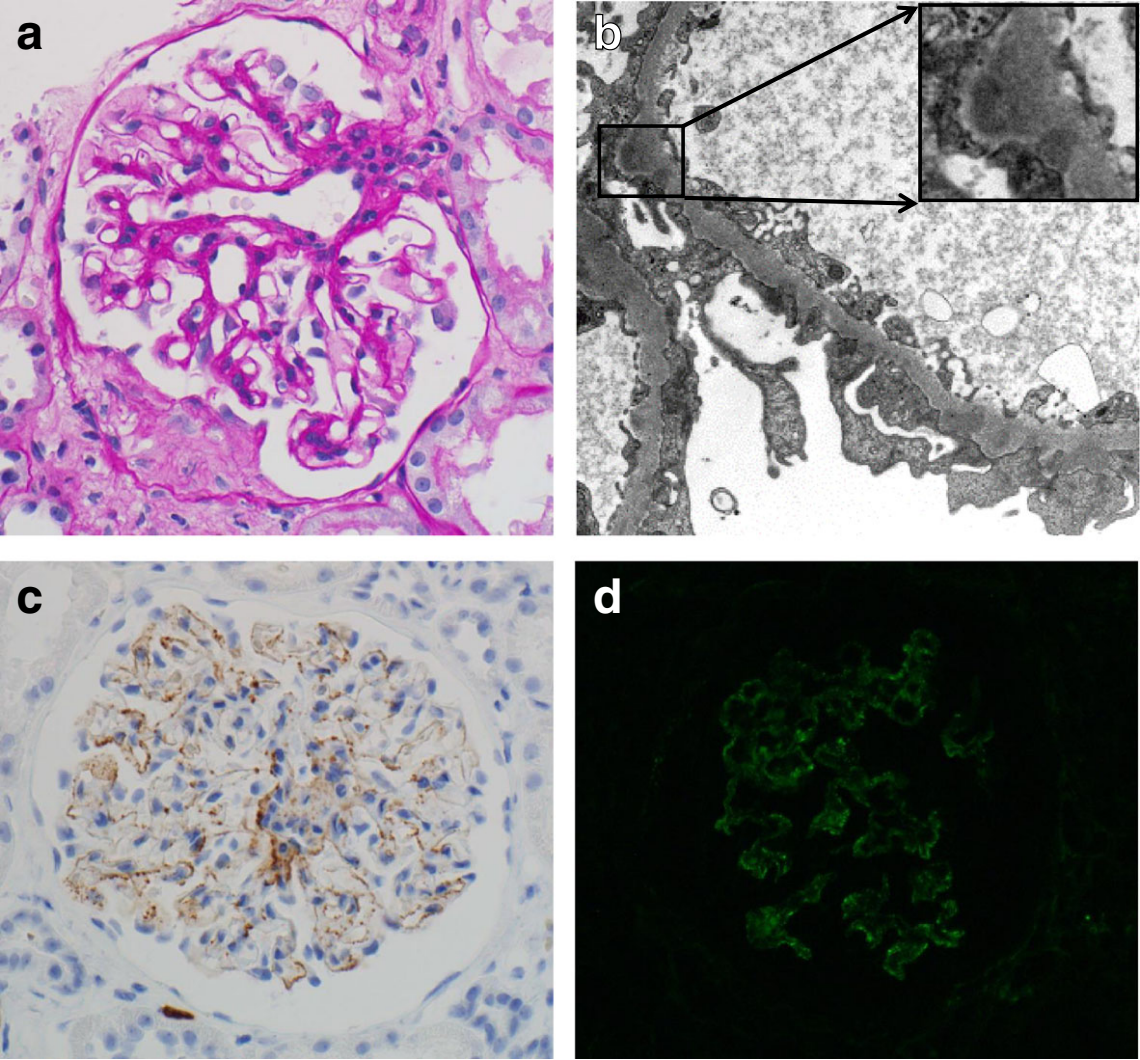
Representative light microscopy, immunofluorescence, and electron microscopy on the biopsy specimen from patient 2. **a** Fibrocellular crescent and thickening and duplication of the glomerular capillary walls (inset) (Periodic acid-Schiff stain). **b** Electron-dense deposits and widespread effacement of podocyte foot processes on electron microscopy. The electron-dense deposits were mainly detected in the subepithelial area, but some were surrounded by the glomerular basement membrane (inset). **c** Immunoperoxidase staining for myeloperoxidase (MPO) showed that the glomerular capillary walls were granularly positive for MPO. **d** Indirect immunofluorescence staining for phospholipase A_2_ receptor (PLA_2_R) showed that the glomerular capillary walls were positive for PLA_2_R. Original magnification: **a**, **c**, **d**: 200 ×

Renal biopsy showed two crescentic glomeruli (Fig. 2a) and two globally sclerotic glomeruli in light microscopy sections containing 19 glomeruli. Diffuse and global spike formation of the glomerular capillary walls was also present. Immunofluorescence staining showed granular 1+ deposition of IgG and complement C3 on the glomerular capillary walls. Electron microscopy revealed subepithelial and intramembranous deposits of high electron density (Fig. 2b). Immunofluorescence staining of IgG subclasses showed deposition of IgG1 and IgG2 but was negative for IgG3 and IgG4. Immunoperoxidase staining showed that the glomerular capillary walls were positive for MPO (Fig. 2c). Immunofluorescence staining of PLA_2_R was positive along the glomerular capillary walls (Fig. 2d), although serum PLA_2_R antibody level at the day of renal biopsy was negative. All findings suggested a diagnosis of MPA, and the renal lesion was diagnosed as ANCA-associated glomerulonephritis (focal class) [7] with MN-lesions.

## Discussion

Although MN and crescentic glomerulonephritis associated with levamisole-adulterated cocaine-induced ANCA-associated vasculitis have been reported [8], ANCA-associated glomerulonephritis and MN are generally considered independent pathologies, and coexistence of the two diseases is thought to be due to chance [9]. However, the common histological findings of the present patients with no history of drug abuse, i.e., both MPO and PLA_2_R in glomerular deposits, suggested that the coexistence of ANCA-associated glomerulonephritis and MN-lesions reflected mutually related diseases rather than a coincidence. There is one reported case of both MPO-ANCA and anti-PLA_2_R antibody positivity in the serum [10], and serum anti-PLA_2_R antibodies and glomerular deposits were simultaneously detected in some patients with ANCA-associated glomerulonephritis combined with MN [11]. To the best of our knowledge, however, these are the first reported cases with ANCA-associated glomerulonephritis and MN-lesions in which both MPO and PLA_2_R were detected in the glomerular deposits.

PLA_2_R staining in the renal tissue was originally reported to be useful for differentiating idiopathic MN from secondary MN [4]. However, glomerular PLA_2_R deposition has since been reported in some patients with secondary MN, such as HBV-, HCV-, and neoplasm-associated MN [12]. Glomerular PLA_2_R was also found to overlap with HBs antigen in patients with HBV-associated MN [5]. In accordance with these findings, the present patients with ANCA-associated glomerulonephritis exhibited glomerular PLA_2_R deposits in MN-lesions.

Matsumoto et al. [13] first reported granular MPO deposition along the glomerular capillary walls in a patient with MPO-ANCA-associated glomerulonephritis complicated by MN-lesions. Hanamura et al. [14] also reported that MPO was detected within electron-dense deposits in patients with MPO-ANCA-associated glomerulonephritis and MN-lesions, and suggested that highly cationic MPO released from activated neutrophils could be trapped by the glomerular basement membrane, thereby forming immune complexes and MN-lesions. This same pathogenic mechanism may be at play in our patients with MN-lesions with persistent positive ANCA titers for MPO. However, it should be kept in mind that because serum anti-PLA_2_R antibodies and tissue PLA2R antigen were not examined in the previous report [14], it cannot be absolutely assumed that MPO is responsible for the MN-lesions.

Anti-PLA_2_R antibodies in the serum have mainly been detected in IgG4, and PLA_2_R and IgG4 are colocalized in the glomerular immune deposits of patients with MN [1]. In addition, IgG1 and IgG2 have been reported to be positive in MPO-ANCA-associated glomerulonephritis and MN-lesions [14]. The finding of positive IgG subclass staining in both patient 1 (all IgG subclasses) and patient 2 (IgG1 and IgG2) was consistent with these previous reports. However, the glomeruli of some patients showed negative IgG4 staining but positive PLA_2_R staining, and the imbalance in the amounts of the corresponding antigens was assumed to influence the results [3]. This explanation could also account for the IgG subclass staining results in patient 2.

Certain inflammatory environments have been suggested to lead to the abnormal expression of PLA_2_R epitopes, resulting in the production of PLA_2_R autoantibodies [12]. Although further studies will be required to elucidate the specific mechanism, we speculate that persistent stimulation by MPO and MPO-ANCA may induce podocyte PLA_2_R expression. Indeed, in patients with both ANCA and anti-glomerular basement membrane (GBM) antibody, it has been suggested that glomerular damage caused by ANCA could expose the target antigen and trigger the production of anti-GBM antibody [15, 16]. However, both of the present cases were negative for serum anti-PLA_2_R antibodies, although the intensity and pattern of PLA_2_R immunostaining were adequate for interpretation as real positivity; PLA_2_R antigen is reportedly expressed on the normal podocyte cytoplasm [2], but was strongly and granularly concentrated within the subepithelial areas in the renal tissue in the present cases (Figs. 1f and 2d). Notably, Debiec and Ronco [17] reported that 10 of 42 patients with primary MN showed positive glomerular PLA_2_R deposition and negative serum PLA_2_R antibodies. As a potential explanation for the discordance between the presence of glomerular PLA_2_R deposition and the absence of serum anti-PLA_2_R antibodies, the authors proposed that the circulating anti-PLA_2_R antibodies are initially deposited in the glomeruli and then rapidly cleared from the blood [17]. Indeed, the rate of patients with positive glomerular PLA_2_R antigens was reported to be much higher than that of patients with positive serum antibodies [17, 18]. It could be also possible that in the present cases without serum anti-PLA_2_R antibodies and IgG4-restricted glomerular deposits, the trapping theory [14] could explain the pathogenesis, and PLA_2_R was only a bystander. In addition, the timing of serum PLA_2_R antibody level measurement could have affected the result, as patient 2 had already been treated with steroid therapy.

## Conclusion

In this report, we described the first cases of individuals diagnosed with ANCA-associated glomerulonephritis with MN-lesions in which both MPO and PLA_2_R were detected in the glomerular deposits. These findings suggest that ANCA-associated glomerulonephritis could expose PLA_2_R, leading to the development of MN-lesions.

## Abbreviations

ANCA: Anti-neutrophil cytoplasmic antibody
IgG: Immunoglobulin G
MN: Membranous nephropathy
MPA: Microscopic polyangiitis
MPO: Myeloperoxidase
PLA_2_R: Phospholipase A_2_ receptor
PR3: Proteinase 3
